# Nursing care of a pediatric patient with anti-NMDAR encephalitis complicated by secondary epilepsy: A case report

**DOI:** 10.1097/MD.0000000000050816

**Published:** 2026-09-18

**Authors:** Qionglei Huang, Luyao Wang

**Affiliations:** a Department of Nursing, Clinical College of Anhui Medical University, Hefei, China.

**Keywords:** anti-NMDAR encephalitis, autoimmune encephalitis, case report, nursing care, pediatrics, secondary epilepsy

## Abstract

**Rationale:**

Autoimmune encephalitis in children often presents with neuropsychiatric symptoms and seizures and may progress to secondary epilepsy with prolonged functional impairment. Immunotherapy is the principal disease-modifying treatment, whereas comprehensive nursing care supports safety, complication prevention, rehabilitation, and continuity of care.

**Patient concerns:**

A 13-year-old girl presented with a 2-month history of intermittent dizziness and poor appetite, followed by abnormal behavior, mood disturbance, visual hallucinations, and seizures.

**Diagnoses:**

Based on characteristic neuropsychiatric manifestations, positive anti-*N*-methyl-d-aspartate receptor antibodies in cerebrospinal fluid and serum, and abnormal electroencephalographic findings, the patient was diagnosed with anti-NMDAR autoimmune encephalitis complicated by secondary epilepsy. Infectious encephalitis was initially considered.

**Interventions:**

The patient received first-line immunotherapy including high-dose corticosteroids and intravenous immunoglobulin, followed by plasma exchange and second-line rituximab therapy. Antiepileptic drugs and anti-infective treatments were administered as indicated. Whole-process nursing care was implemented across acute, intensive care, rehabilitation, and post-discharge phases.

**Outcomes:**

After multidisciplinary treatment and nursing support, the patient regained clear consciousness with improved cognition and communication. Lower-limb muscle strength improved to grade 4/5, seizure frequency markedly decreased, and independent ambulation was achieved. At 3-month follow-up, no relapse was reported and the patient had returned to school.

**Lessons:**

This case highlights the importance of structured seizure safety management, strict infection prevention during immunotherapy, individualized rehabilitation guidance, and family-centered continuing care in pediatric anti-NMDAR autoimmune encephalitis complicated by secondary epilepsy.

## 1. Introduction

Autoimmune encephalitis (AE) comprises inflammatory encephalitis syndromes mediated by autoimmune mechanisms and is increasingly recognized in children.^[1]^ Anti-*N*-methyl-d-aspartate receptor (NMDAR) encephalitis is the most frequently identified antibody-mediated AE in the pediatric population.^[2,3]^ Seizures are common in pediatric AE and may be an early or dominant manifestation; a proportion of patients progress to status epilepticus, particularly younger children.^[4]^ Secondary epilepsy refers to recurrent seizures arising from an underlying structural or functional brain disturbance and may complicate the disease course and recovery.^[5]^ Given the complex neuropsychiatric presentation, risk of seizures and complications, and prolonged rehabilitation needs, whole-process nursing care is essential but underreported in the pediatric AE literature. Here we report the diagnosis, treatment course, and nursing care of a 13-year-old girl with anti-NMDAR AE complicated by secondary epilepsy, emphasizing nursing priorities across acute care, intensive care, rehabilitation, and continuing care.

## 2. Case presentation

### 2.1. General information

A 13-year-old girl was admitted after a disease course characterized by intermittent dizziness, poor appetite, and progressive neuropsychiatric symptoms. According to her caregiver, the earliest manifestations had begun approximately 2 months before the initial hospitalization. She subsequently experienced episodes of inappropriate laughter lasting several minutes, confusion, incoherent speech, and visual hallucinations.

The patient and her parents denied significant past medical history. There was no family history of major neurologic or psychiatric illness. There was no history of head trauma, substance use, or exposure suggestive of intoxication. The family denied domestic violence, physical abuse, or sexual assault.

### 2.2. Clinical findings

On examination, vital signs were stable (temperature 36.2°C, pulse 76 beats/min, respiration 20 breaths/min, blood pressure 126/65 mm Hg). Her weight was 73 kg. She had Cushingoid facies and acneiform eruptions, which were considered most likely related to corticosteroid exposure during treatment.

Neurologic examination showed slightly decreased knee reflexes. Lower-limb muscle strength was grade 4/5; she could walk independently but had difficulty descending stairs and could not run or jump quickly. At the time of assessment in our department, she was oriented and responsive, with clearer thinking and improved memory compared with earlier assessments.

Cerebrospinal fluid testing was positive for anti-NMDAR antibodies (1:32), and serum antibodies were also detected (1:10).^[6,7]^ Video electroencephalogram (EEG) showed epileptiform abnormalities, including sharp and slow waves predominantly in the left temporal-occipital regions with additional discharges in frontal and midline regions, supporting ongoing seizure susceptibility.^[8]^ The detailed clinical findings and laboratory results are summarized in Table 1.

**Table 1:** Timeline of key events, treatments, and outcomes.

Date/phase	Key events	Key treatments	Key outcomes/nursing focus
Jun–Sep 2024 (Onset)	Headache, poor appetite; neuropsychiatric symptoms (laughter, hallucinations, confusion)	Initial assessment; infectious encephalitis considered	Early recognition of neuropsychiatric change; safety and caregiver education
Sep 30, 2024	Seizures occurred	Levetiracetam initiated	Seizure precautions; documentation of semiology and triggers
Oct 8, 2024 (PICU transfer)	Clinical deterioration with seizures/infection concerns	Escalated antiepileptic and anti-infective management	Airway protection; neurologic monitoring; infection surveillance
Oct 25–Nov 15, 2024	Refractory course requiring second-line immunotherapy	Rituximab 500 mg/dose (4 doses across Oct 25–Nov 15)	Medication safety monitoring; psychosocial support
Nov 9–Nov 24, 2024	Severe course requiring adjunctive therapy	Plasma exchange (8 sessions)	Device care/asepsis; monitoring for complications; caregiver communication
Nov 29–Dec 17, 2024 (ward/rehabilitation)	Admitted to our department for ongoing care and rehabilitation	Supportive care; oral prednisone taper; continued antiepileptic therapy	Strength and mobility training; swallowing support; discharge planning and continuing care
3 mo post-discharge	Follow-up by telephone/online communication	Adherence reinforcement; relapse surveillance	No relapse; returned to school; ongoing rehabilitation guidance

PICU = pediatric intensive care unit.

### 2.3. Diagnosis

Based on the acute neuropsychiatric symptoms, seizures, abnormal EEG findings, and positive anti-NMDAR antibodies in cerebrospinal fluid and serum, the patient was diagnosed with anti-NMDAR AE complicated by secondary epilepsy. At presentation, infectious encephalitis was also considered given altered mental status and systemic risk; therefore, empiric anti-infective therapy was initiated while diagnostic evaluation progressed.^[1,2]^ Differential diagnoses also included toxic/metabolic encephalopathy and primary psychiatric disorders. Toxic ingestion was less likely given the absence of exposure history or substance use. The detection of anti-NMDAR antibodies, together with the characteristic neuropsychiatric manifestations and seizure evolution, supported AE as the unifying diagnosis.^[1,2]^

### 2.4. Therapeutic interventions and nursing process

Initial management included anti-infective therapy (ceftriaxone plus ganciclovir), immunotherapy with high-dose methylprednisolone and intravenous immunoglobulin, and supportive measures for intracranial pressure control, alongside symptomatic treatment.

During the subsequent course, seizures occurred and levetiracetam was initiated. After urinalysis suggested fungal infection, the regimen was adjusted to cefoperazone/sulbactam plus acyclovir, with fluconazole for antifungal coverage. Because of worsening consciousness disturbance, recurrent fever, and seizure aggravation, the patient was transferred to the pediatric intensive care unit, where plasma exchange was performed and antimicrobial therapy was repeatedly adjusted. Antiepileptic treatment during the critical phase included midazolam, sodium valproate, and levetiracetam. Following a peripherally inserted central catheter-line blood culture indicating Staphylococcus epidermidis, antibiotics were adjusted to cefoperazone/sulbactam plus vancomycin and later switched to linezolid. After blood testing indicated human herpesvirus type I, acyclovir was reintroduced, and broad-spectrum antibiotics were used for infection management. Second-line immunotherapy with rituximab was initiated during the later treatment phase, with additional doses administered after transfer back to our department, followed by oral prednisone acetate 55 mg. The timeline of major treatments and nursing interventions is summarized in Table 2.

**Table 2:** Major therapeutic components and rationale (summary).

Domain	Interventions (examples)	Rationale	Nursing considerations
Immunotherapy	High-dose corticosteroids; IVIG; plasma exchange; rituximab	Control immune-mediated inflammation; treat refractory disease	Monitor vitals/glucose/BP; infection prevention; caregiver education on adherence and adverse effects
Seizure control	Levetiracetam; additional antiepileptic agents as needed; rescue therapy	Prevent recurrent seizures/status epilepticus and secondary injury	Seizure precautions; airway protection; record semiology; ensure timely medication administration
Anti-infective management	Empiric antibacterial/antiviral therapy; regimen adjustment based on cultures/clinical course	Address suspected/confirmed infections during immunomodulation and device use	Aseptic technique; catheter care; early recognition of fever/local signs; antimicrobial safety monitoring
Rehabilitation/continuing care	Mobility, gait/balance and swallowing training; family-centered discharge education; follow-up	Promote functional recovery and safe reintegration	Fall prevention; safe feeding; motivational support; structured follow-up for relapse surveillance

IVIG = intravenous immunoglobulin.

### 2.5. Outcomes and follow-up

Post-discharge follow-up was arranged by pediatric nurses via telephone 1 week after discharge to identify early warning symptoms and support adherence. Nurses maintained a personalized health record and provided a written nursing manual covering medication precautions, diet and activity recommendations, daily safety, and home-environment guidance. An online communication channel (e.g., WeChat group) was used to share disease-related information, receive caregiver feedback, and provide timely long-term support.

At discharge from our department, the patient was conscious with appropriate responses and improved cognition compared with the acute phase. Lower-limb muscle strength was grade 4/5; she could ambulate independently and descend stairs with mild difficulty, but she could not run or jump. Neurologic examination documented recovery of knee reflexes to normal, and caregivers reported a marked reduction in seizure frequency with continued antiepileptic therapy. At 3-month follow-up after discharge, no relapse was reported and the child had returned to school.

## 3. Nursing

### 3.1. Neurologic monitoring and seizure safety

Given the risk of rapid deterioration in AE with secondary epilepsy, nurses conducted scheduled and event-triggered monitoring of vital signs and neurologic status (consciousness, behavior, speech, pupillary response, and motor function), documenting deviations and escalating promptly to support treatment adjustments.^[9,10]^ Seizure precautions were maintained, and emergency equipment and prescribed rescue medications were kept readily available.

During seizures, nurses prioritized airway protection and injury prevention by positioning the child safely with the head turned to 1 side, providing oxygen as needed, raising bedrails, and avoiding forceful limb restraint. Seizure features (onset, duration, semiology, triggers, and recovery) were recorded to inform subsequent management.^[9,11]^ Nurses also monitored for warning signs of raised intracranial pressure or impending herniation (declining consciousness, pupillary changes, persistent vomiting, severe headache, agitation) and escalated urgently when present.^[9,10]^

### 3.2. Medication safety and monitoring

Nurses ensured accurate administration of antiepileptic and immunomodulatory therapies, reinforced adherence, and warned caregivers against unsupervised steroid dose changes. For intravenous antivirals and other infusions, infusion rates were controlled per orders to minimize irritation and maintain hemodynamic stability and fluid-electrolyte balance. Caregivers were advised to complete scheduled monitoring (blood counts, liver/renal function, glucose, and blood pressure) during follow-up to support safety during prolonged treatment.^[12]^

### 3.3. Infection prevention and device-related care

Recurrent infections occurred during immunotherapy and invasive support. Nurses implemented strict aseptic technique, performed routine surveillance for infection indicators (temperature trends, urinary symptoms, catheter-site changes), and coordinated timely reassessment and antimicrobial adjustments with clinicians. For urinary tract infections, nursing care emphasized perineal hygiene, adequate hydration, and medication adherence, which supported symptom management.^[12]^

### 3.4. Rehabilitation, swallowing support, and family-centered care

At admission, the child showed slightly diminished knee reflexes and grade IV lower-limb muscle strength, consistent with motor dysfunction reported in AE. Nursing assessment of mobility and fall risk supported individualized rehabilitation planning. Stepwise strengthening, gait and balance training, range-of-motion exercises, and caregiver coaching were provided, with positive reinforcement to promote engagement.^[13]^ Swallowing difficulty was addressed through safe-feeding strategies and coordinated rehabilitation (e.g., oral motor and sensory training) to reduce aspiration risk.^[13]^

Psychosocial care targeted distress related to prolonged hospitalization and steroid-associated appearance changes. Nurses provided anticipatory guidance, emotional support, and practical advice, and continuing care after discharge (telephone follow-ups and scheduled reviews) reinforced medication adherence, rehabilitation goals, and relapse surveillance^.[10,14]^

## 4. Discussion

Clinical practice guidelines and expert consensus offer standardized frameworks for diagnosis, treatment, and supportive care, reducing practice variation and enabling evidence-based decisions. In this case, management broadly aligned with current recommendations for AE and seizure care. Immunotherapy remained the principal disease-modifying treatment, whereas nursing practice emphasized early detection of deterioration, prevention of secondary injury, infection control during immunotherapy, and coordinated rehabilitation to support longer-term recovery.^[15-17]^

A key implication of this case is the value of nursing surveillance and clinical reasoning in complex pediatric neuroimmunologic illness. Structured neurologic assessments, readiness for seizure rescue, and timely escalation may reduce avoidable complications such as aspiration, trauma, and delayed intervention, while providing actionable bedside information for medical decision-making.^[9]^ The EEG abnormalities and recurrent seizure activity in this patient were interpreted together with the overall encephalitic process rather than in isolation, underscoring the importance of longitudinal observation and follow-up.

Infectious complications can complicate neurologic recovery during immunomodulation and invasive support. Rigorous aseptic practice, early recognition of infection, adherence support for antimicrobials, and caregiver education are essential, particularly in children with nonspecific early symptoms.^[12]^ Functional recovery likewise requires sustained rehabilitation and family engagement. Individualized mobility and swallowing training, combined with caregiver coaching and motivational support, may facilitate reintegration and improve quality of life.^[13]^ Psychosocial support addressing distress and appearance changes further strengthens holistic recovery and caregiver coping, and structured continuing care supports adherence and relapse monitoring after discharge.^[9,13,14]^

## 5. Conclusion

This case highlights that pediatric anti-NMDAR AE complicated by secondary epilepsy requires whole-process nursing care across acute management, intensive monitoring, rehabilitation, and continuing care. Structured seizure safety management, strict infection prevention during immunotherapy and invasive-device care, progressive functional rehabilitation, and family-centered support were important to safe care delivery and discharge readiness.

## 6. Limitations

This report describes a single patient; therefore, the findings may not be generalizable. Several objective clinical outcomes were available, including muscle strength, ambulation, and school reintegration; however, standardized functional or cognitive scales were not documented. In addition, because this case occurred some time ago and the original records could not be fully retrieved, neuroimaging findings (MRI/CT) and tumor-screening information were unavailable for inclusion. These limitations notwithstanding, the case provides practical nursing insights into seizure safety, infection prevention, rehabilitation support, and continuing care for pediatric anti-NMDAR AE with secondary epilepsy.

## Acknowledgments

The authors thank the patient and her legal guardian for consenting to the publication of this case.

## Author contributions

**Methodology:** Luyao Wang.

**Writing – original draft:** Qionglei Huang.
